# Prevalence of Middle Mesial Canal in Mandibular First Permanent Molars in a Persian Population: An In Vivo Cone‐Beam Computed Tomography Study

**DOI:** 10.1002/cre2.935

**Published:** 2024-08-28

**Authors:** Samaneh Hajizadeh, Morteza Khodabandeh Amiri, Sanaz Mihandoust, Zhaleh Shafiei Sabet, Rasoul Tabari Khomeiran

**Affiliations:** ^1^ Department of Endodontics, School of Dentistry Guilan University of Medical Sciences Rasht Iran; ^2^ School of Dentistry, Dental Sciences Research Center Guilan University of Medical Sciences Rasht Iran; ^3^ Department of Dental and Maxillofacial Radiology, School of Dentistry Guilan University of Medical Sciences Rasht Iran; ^4^ Department of Nursing, Social Determinants of Health Research Center (SDHRC) Guilan University of Medical Sciences Rasht Iran

**Keywords:** cone‐beam computed tomography, mandibular first molar, middle mesial canal, root canal therapy

## Abstract

**Objectives:**

This study aimed to identify the prevalence of the middle mesial canal (MMC) in mandibular first permanent molars in a Persian subpopulation sample using cone‐beam computed tomography (CBCT). The secondary aim was to correlate the incidence of MMC with variables such as gender, age, and the presence of an additional distal canal.

**Materials and Method:**

A total of 390 mandibular first molars from 314 CBCT images were retrospectively evaluated. The presence of the MMC was recorded while considering related factors such as additional distal canal and demographic information of the patients (age and gender). Fisher's exact tests and independent‐samples *t*‐test were used for the statistical comparisons with a significance level of 0.05.

**Results:**

Among 390 mandibular first molars, 34 teeth contained an MMC (8.7%). The number of teeth with the MMC in women was equal to that of men. There was no significant difference between the two genders in the prevalence of the MMC (*p* > 0.05). The prevalence of patients' MMC had no significant relationship with their age (*p* > 0.05). However, there was a significant association between the presence of the MMC and an additional distal canal (*p* < 0.05).

**Conclusion:**

The incidence of an additional distal canal in teeth with a diagnosed MMC was statistically significant. The prevalence of the MMC in mandibular first molars in the study population was low; however, accurate knowledge of root canal morphology in terms of the presence of an additional root canal in these teeth should be considered.

## 1 Introduction

Successful endodontic treatment requires clinicians to have sufficient knowledge of root morphology, which enables them to thoroughly remove irritants such as necrotic pulp tissue, microorganisms, and their byproducts from the root canal system (RCS) (Aldosimani et al. 2021; Weinberg et al. 2020; Versiani et al. 2022). The complex anatomy of RCS, in some cases, may lead to failure in detecting the canal, thus interfering with the proper cleaning, shaping, and sealing of RCS (Yang et al. 2020). Missing a root canal in failed endodontic treatment has been reported in 23.04% of cases (Berman and Hargreaves 2020). The mandibular first molar (MFM) is the first permanent tooth to erupt. Pits and fissures on these teeth may cause early caries, leading to a higher chance of needing endodontic treatment (Qiao et al. 2020; Silva et al. 2013). The most common form of MFM has generally been described as having a mesial root containing a mesiobuccal and a mesiolingual canal and a distal root often containing one canal (Aldosimani et al. 2021; Iqbal, Kochhar, and Kumari 2022). However, aberrant root canal morphologies are not uncommon in the mandibular permanent molars (Aldosimani et al. 2021). The presence of middle mesial canal (MMC) in these teeth was first reported by Vertucci and Williams in 1974 (Vertucci and Williams 1974). Pomeranz classified the MMC into three types: fin, confluent, and independent. The fin type does not have a separate orifice, while the file can move between the MMC and the mesiobuccal or mesiolingual canals. The confluent type has a separate orifice and only joins the mesiobuccal or mesiolingual canal in the apical area. On the other hand, the independent type has both the orifice and apical foramen separately (Pomeranz, Eidelman, and Goldberg 1981; Bhatti et al. 2022).

The overall prevalence of MMC in MFMs is estimated to be 7% worldwide, ranging from 1% to 23% in different countries (Pertek Hatipoğlu et al. 2023). MMC can be found within the isthmus area, a narrow connection between the two mesial root canals (Kuzekanani, Walsh, and Amiri 2020). Detecting and accessing MMCs is reported to be challenging, and untreated MMCS can lead to endodontic treatment failure due to inadequate instrumentation and disinfection (Bhatti et al. 2022; Chavda and Garg 2016; Shah and Khan 2019). Hence, it is crucial to detect and disinfect any anatomical variations, such as MMC and an additional distal canal, to ensure successful nonsurgical root canal treatment. Moreover, some studies have shown that the presence of MMC and an additional distal canal may be correlated (Sherwani et al. 2016; Nosrat et al. 2015).

Therefore, the current study aimed to evaluate the prevalence of MMC in mandibular first permanent molars among the Guilanian population, a Persian subpopulation living in Guilan province, Northern Iran, and to investigate the relationship between MMC and patient demographics using cone‐beam computed tomography (CBCT) to provide clinicians with detailed anatomical imaging data and to improve the success rate of endodontic treatment. The secondary aim was to investigate if there is a significant correlation between MMC and the presence of an additional distal canal. In case of the existence of such a correlation, it is essential to carefully examine the presence of an additional distal canal in MFMs with MMC.

## 2 Materials and Methods

This study was approved by the Research Ethics Committee of Guilan University of Medical Sciences (approval ID: IR.GUMS.REC.1400.619) and was conducted in accordance with the Declaration of Helsinki.

### 2.1 Study Design

In this analytical cross‐sectional study, CBCT images of 314 patients were randomly selected from the archives of the Oral and Maxillofacial Radiology Department of Guilan Dentistry School, taken between 2016 and 2022, for different diagnostic assessment tasks. The inclusion criteria were as follows:

1. CBCT images of patients over 12 years of age
2. Containing at least one MFM
3. MFMs with closed apex
4. MFMs without any root fracture
5. MFMs without previous endodontic treatment
6. MFMs without calcified pulp chambers or canals
7. MFMs without root resorption
8. High‐quality CBCT images without any artifacts

The minimum sample size, based on Shakeri et al. (2019), was estimated to be 314 teeth using the sample size calculation formula

$$N=\frac{Z^{2}\times P\times \left(1-P\right)}{d^{2}},$$

where *Z* for a 95% confidence level is 1.962, the prevalence of the MMC (*P*) is 0.0338, *d* is the precision limit, and $N=\frac{1.96^{2}\times 0.0338\times \left(1-0.0338\right)}{0.02^{2}}≃314$.

A total number of 390 teeth were investigated in 314 patients. The included sample size was larger than the minimal size to promote the external validity of the study.

### 2.2 Radiographic Evaluation

CBCT images were acquired by Pax‐i3D device (Vatech, Yongin, Korea) at 95 kV, 5.2 mA, FOV 150 mm × 150 mm, and voxel size of 0.2 mm. For intra‐examiner calibration, the examiner was trained by a radiologist on how to evaluate the samples before starting the analysis of the CBCT images. The examiner evaluated the presence or absence of the MMC using Ez3D‐i software (Vatech, Korea) on a 22‐in. SAMSUNG LED monitor with a resolution of 1920 × 1080, from the axial plane by moving from the crown area to the apex area. A radiologist re‐evaluated 50% of the samples for interobserver reliability on the same monitor. The position of the tooth in the jaw, the presence of an additional distal canal, and the distribution of age and gender were investigated.

### 2.3 Statistical Analysis

For descriptive statistical analysis, absolute and relative frequency were used, and the Fisher's exact tests and independent‐samples *t*‐test were applied to compare differences. SPSS software, version 21 (IBM, Armonk, NY, USA), was used for statistical analysis. The level of statistical significance was set at 0.05 (*p* < 0.05).

## 3 Results

The study examined CBCT images of 390 MFMs from 314 patients, with an average age of 38.76 ± 14.06 years, including 164 females (52.20%) and 150 males (47.80%). Among the 390 MFMs examined, 34 teeth (8.70%) had MMC. A higher proportion of the evaluated teeth were on the right side (58.50%). Consequently, over 50% of the teeth with MMC were on the right side (52.90%).

More than 50% of the assessed teeth (58.50%) and teeth with MMC (52.90%) were on the right side.

**Figure 1:**
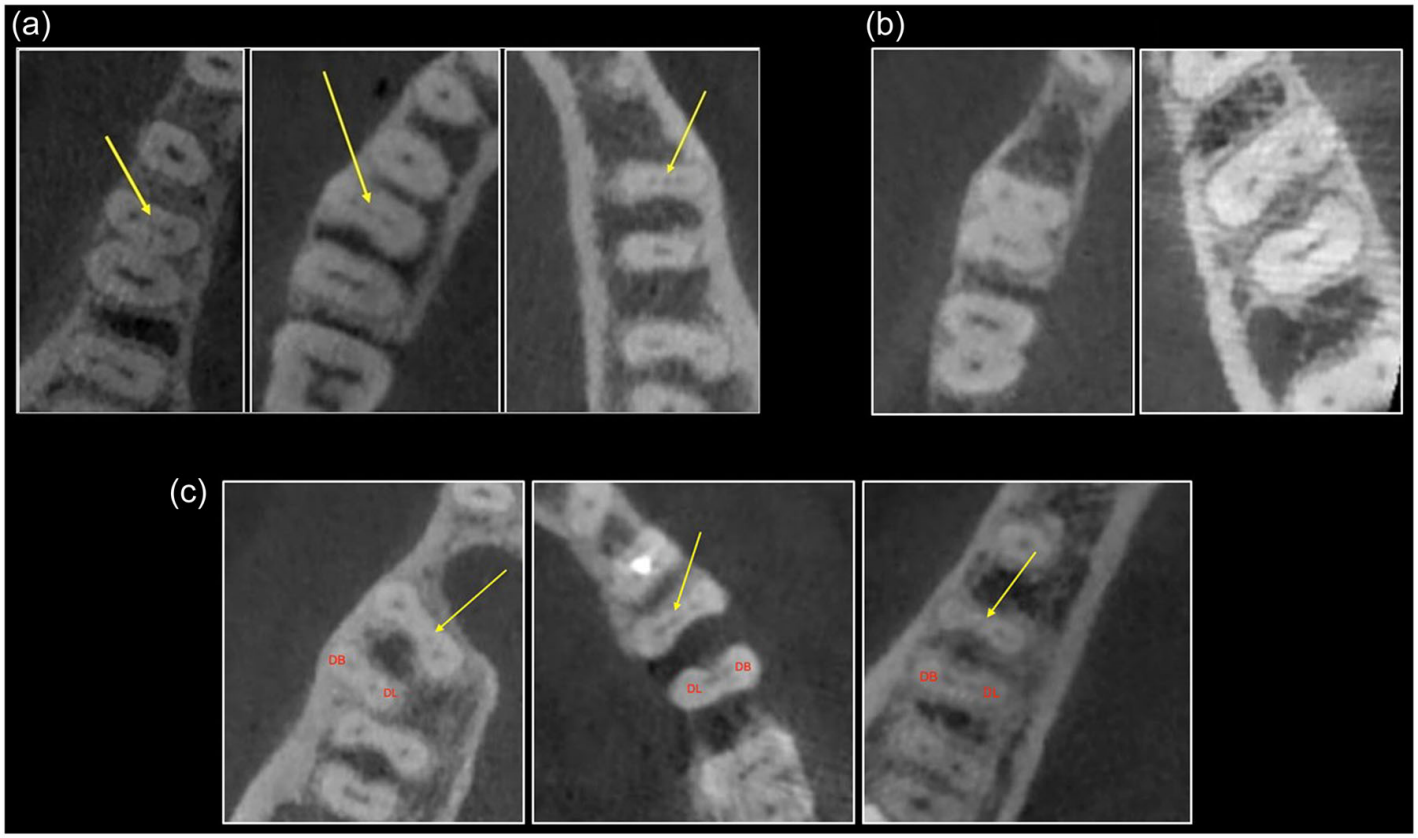
(a) Axial view of the CBCT images with middle mesial canal, (b) axial view of the CBCT images without middle mesial canal, and (c) axial view of the CBCT images with middle mesial canal and an additional distal canal. The yellow arrows point to the middle mesial canal. DB, distobuccal; DL, distolingual.

Table 1 demonstrates the distribution of MMC based on age, gender, and the presence of an additional distal canal. The result of the independent‐samples *t*‐test indicates no significant relationship between the prevalence of MMC and the age (*p* > 0.05). Fisher's exact test indicated that there was no significant relationship between gender and the prevalence of MMC (*p* > 0.05). Hence, the number of teeth with MMC in men and women was equal (17 cases in each gender). In 55.9% of the MFMs with MMC, an additional distal canal was also observed; however, the prevalence of an additional distal canal among all examined teeth was 31.3% (Figure 1). Fisher's exact test indicated a significant relationship between the simultaneous presence of MMC and the additional distal canal (*p* < 0.05).

**Table 1:** Distribution of middle mesial canal in mandibular first molars by age, gender, and the presence of additional distal canal.

	Age, mean ± SD (N*)	Males, *n* (%)	Females, *n* (%)	Additional distal canal present (%)	Additional distal canal absent (%)
MMC present	38.65 ± 12.03 (34)	17 (50)	17 (50)	19 (55.9)	15 (44.1)
MMC absent	38.78 ± 14.26 (356)	163 (45.8)	193 (54.2)	103 (28.9)	253 (71.7)
Total	38.76 ± 14.06	180 (46.2)	210 (53.8)	122 (31.3)	268 (68.7)
*p*	0.958	0.720		0.003	

Abbreviations: MMC, middle mesial canal; N*, number.

## 4 Discussion

Posterior teeth including MFMs are among the most difficult teeth in root canal treatment due to the complex and varied morphology of their root canal system. Over the past few years, researchers have conducted numerous studies to explore the anatomical structure and the number of accessory root canals using different methods. However, a few studies examined the prevalence and variations of MMC in MFMs using CBCT imaging. According to Kim, Lee, and Woo (2012), CBCT scans could make the morphology of the root canal system more understandable and cause improvements in the outcome of endodontic treatments. The accuracy of CBCT imaging for detecting the second mesiobuccal canal in maxillary molars has been reported to be 96% (Mirmohammadi et al. 2015).

Hence, the current study used CBCT imaging for the detection of MMC in MFMs. In this study, the prevalence of MMC in MFMs was 8.7%, which is in agreement with the studies conducted by Hosseini et al. (2020) and Kuzekanani, Walsh, and Amiri (2020). The prevalence of MMC in MFMs is reported to be within a wide range of 1.3%–27% in various studies (Kuzekanani, Walsh, and Amiri 2020; Shakeri et al. 2019; Hosseini et al. 2020; Hu et al. 2019; Akbarzadeh et al. 2017; Soares de Toubes et al. 2012; Srivastava, Alrogaibah, and Aljarbou 2018). Table 2 provides a summary of similar studies that evaluated the prevalence of the MMC in MFMs using CBCT images. The variation in the reported results could be attributed to factors such as ethnicity, sample size, age, and the study's methodology, including the techniques used (clinical or CBCT) (Bhatti et al. 2022; Ingle, Bakland, and Baumgartner 2008). The higher reported prevalence of MMC, as obtained by Akbarzadeh et al. (2017) or Soares de Toubes et al. (2012), could be justified by ethnic variations, age, or methodological differences. In a multicenter study conducted on populations from Brazil and Turkey, it was shown that ethnicity can be the cause of differences in the prevalence of MMC. Therefore, multicenter and clinical research is needed in the future to determine the relationship between the ethnic and the prevalence of MMC to improve endodontic treatments (Versiani et al. 2016). Moreover, the results obtained from studies evaluating the presence of MMC with clinical techniques are in the higher range of 20%–46.2% (Chavda and Garg 2016; Nosrat et al. 2015; Azim, Deutsch, and Solomon 2015). The low frequency of MMC identified in the present study can be attributed to the age of the evaluated population and the use of CBCT for detection, as both factors are proven to affect MMC identification (Nosrat et al. 2015). Methodological differences can also cause varying reports of the frequency of MMC, depending on whether the observation unit is based on the patient (Shah and Khan 2019; Akbarzadeh et al. 2017) or the second molar tooth (Hu et al. 2019; Tahmasbi et al. 2017). Additionally, different spatial resolutions of CBCT appliances associated with varied slice thickness could affect the observer's ability to identify the MMC and consequently cause various reported frequencies of this canal; however, most previous studies have not mentioned the resolution within their studies (Table 2).

**Table 2:** Previous studies evaluating the frequency of middle mesial canal in mandibular first molars.

Author	Location	Number of mandibular first molars (*n*)	MMC (%)	CBCT detector resolution (pixel)
Aldosimani et al. (2021)	Saudi Arabia	1377 (mandibular first and second molars)	1.3	N/A
Yang et al. (2020)	China	1750	9.03	1024 × 1024
Bhatti et al. (2022)	Islamabad, Pakistan	298	7.7	N/A
Kuzekanani, Walsh, and Amiri (2020)	Kerman, Iran	100	8.1 6.3 M 10.0 F	N/A
Shakeri et al. (2019)	Tehran, Iran	207	2.5 M 4 F	N/A
Hosseini et al. (2020)	Babol, Iran	200	9	N/A
Hu et al. (2019)	Guangzhou, China	823	10.8	N/A
Akbarzadeh et al. (2017)	Ohio, USA	210	14.7	N/A
Soares de Toubes et al. (2012)	Brazil	44 (extracted teeth)	27.0	N/A
Srivastava, Alrogaibah, and Aljarbou (2018)	Saudi Arabia	143	18.2	N/A
Versiani et al. (2016)	Brazil	136	22.1	N/A
	Turkey	122	14.8	
Nosrat et al. (2015)	Baltimore, MD	75 (mandibular first and second molars)	20 (mandibular first and second molars)	N/A
Tahmasbi et al. (2017)	Texas, USA	122 (mandibular first and second molars)	26	N/A

Abbreviations: MMC, middle mesial canal; N/A, not applicable.

In the present study, no significant relationship was found between age and the prevalence of MMC, which is similar to a study conducted in Saudi Arabia, reporting the absence of a significant relationship between the prevalence of MMC (1.3%) and age (Aldosimani et al. 2021). Another study carried out by Tahmasabi et al. (2017) in Texas, USA, reported no significant relationship between the prevalence of MMC in MFMs, which was 26%, and the age of participants. However, some studies have shown that it is easier to identify additional root canals in younger patients because the rate of physiological or pathological calcification of the pulp is reported to be lower in younger patients compared to older patients (Nosrat et al. 2015; Azim, Deutsch, and Solomon 2015; Karapinar‐Kazandag, Basrani, and Friedman 2010). In contrast, Hu et al. (2019) concluded that the prevalence of the MMC was associated with age, as this prevalence was significantly higher in 41‐ to 60‐year‐old patients in comparison to the ≤ 20‐year‐old patients. According to the study mentioned, the prevalence of MMC is higher in older patients because of the secondary dentin deposition in the mesiodistal direction of the root canals. This results in the division of a large canal into two more complex and separated canals (Hu et al. 2019). Similarly, Srivastava, Alrogaibah, and Aljarbou (2018) reported that the prevalence of MMC was significantly higher in 30‐ to 60‐year‐old patients due to the secondary dentin deposition during this transition period for root canal differentiation.

Furthermore, the current study showed that there is no significant relationship between gender and the prevalence of MMC. Other studies such as Yang et al.'s (2020) in China and Tahmasabi et al.'s (2017) conducted in Texas have also reported the absence of a significant relationship between gender and the prevalence of MMC. However, in the study of Kuzekanani, Walsh, and Amiri (2020) in Iran, Kerman, the prevalence of MMC was reported to be much higher in women compared to men. However, the examined sample size in the mentioned study is small, and consequently, the results may not have sufficient validity.

In our study, 55.9% of the MFMs with MMC had an additional distal canal. Fisher's exact test indicated a significant relationship between the presence of the mentioned two additional canals. This clinically significant finding is in agreement with the Sherwani et al. (2016) study, which reported a significant relationship between the occurrence of MMC and the presence of two distal canals (*p* < 0.05). Two distal canals were present in 74% (54/73) of teeth with an MMC in their study. Among the teeth without MMC, only 35.1% (65/185) had two distal canals. These statistics are in accordance with our results (Table 2). In contrast, Nosrat et al. (2015) found no significant association between MMC and the presence of a second distal canal. The examined sample size in the study done by Nosrat et al. (2015) was only 75 mandibular molars, which was less than our study (390 MFMs) and the study by Sherwani et al. (2016) (258 MFMs). In our study and the study by Sherwani et al. (2016), only MFMs were investigated, while Nosrat et al. (2015) evaluated both mandibular first and second molars, which can cause different results.

## 5 Conclusion

According to the results of this study, the prevalence of MMC in MFMs is low, but the clinician must consider the possibility of its existence and search for finding it so that he could do a complete and successful root canal treatment. The presence of an additional distal canal should also be considered in MFMs with MMC.

## Author Contributions

**Samaneh Hajizadeh:** conceptualization, methodology, writing–original draft. **Morteza Khodabandeh Amiri:** methodology, visualization, writing–review and editing. **Sanaz Mihandoust:** methodology, visualization, writing–original draft, writing–review and editing. **Zhaleh Shafiei Sabet:** methodology, visualization, writing–review and editing. **Rasoul Tabari Khomeiran:** statistical analysis, writing–review and editing.

## Conflicts of Interest

The authors declare no conflicts of interest.

## Data Availability

The data that support the findings of this study are available from the corresponding author upon reasonable request.
